# Factors associated with medical students’ interest in remote and very remote practice in Australia: A national study

**DOI:** 10.1111/ajr.12694

**Published:** 2021-02-08

**Authors:** Dylan Raftery, Vivian Isaac, Lucie Walters

**Affiliations:** ^1^ Flinders University School of Medicine Bedford Park SA Australia; ^2^ Flinders Rural Health South Australia Flinders University Renmark SA Australia; ^3^ Adelaide Rural Clinical School University of Adelaide Mount Gambier SA Australia

**Keywords:** career intent, medical school, rural clinical school, rural health, rural workforce shortage

## Abstract

**Objective:**

To investigate the factors that are associated with medical student interest in remote and very remote practice in Australia.

**Design:**

Aggregated data of an annual cross‐sectional survey from 2013 to 2017.

**Setting:**

Australia.

**Participants:**

Medical students from 17 medical schools, at the point of finishing one year of clinical training in a rural or remote location in Australia.

**Main outcome measures:**

Intention for working in a remote or very remote location as a doctor.

**Results:**

Responses were analysed from 3328 medical students. From this cohort, 37.6%, 54.0% and 7.0% of students reported future career intent in capital or major cities; regional Australia; and remote or very remote Australia respectively. Multivariable analysis indicated students interested in remote and very remote practice compared to those interested in regional practice were more likely to be from a rural background, have prior generalist intentions, felt as though their rural clinical school (RCS) experience increased interest in remote and very remote practice, and had higher rural practice self‐efficacy. Odds ratios were larger for these factors when students interested in remote or very remote practice were compared with students interested in practicing in capital or major cities.

**Conclusions:**

Rural background, prior generalist intentions, rural practice self‐efficacy and the overall influence of the RCS experience are associated with interest in remote and very remote practice.

**What is already known on this subject?**

- Rural career intent is associated with rural background, prior generalist intentions, long rural clinical placements, satisfaction with these placements and positive wellbeing during these placements
- Previous Australian studies have classed rural broadly. Previous research has not differentiated rural from remote and very remote areas in Australia, where workforce shortages are the largest

**What does this study add?**

- Many of the previous associations with rural career intent hold true for remote and very remote career intent
- Rural placement satisfaction is not associated with remote and very remote career intent
- This study affirms the recent Rural Health Multidisciplinary Training Evaluation recommendation to increase remote clinical placements

## 1 INTRODUCTION

Australia is still suffering from a medical workforce shortage. While this shortage is reflected throughout all areas outside of capital or major cities, it is particularly prominent in remote and very remote areas despite their greater need for these services.^1^, ^2^

Understanding what makes doctors more likely to practise in remote and very remote areas will allow us to try and reduce this shortage. It is well researched in the literature that students with a rural background are more than twice as likely to practise rurally than their urban counterparts.^3^, ^4^, ^5^, ^6^ Multiple large‐scale studies have also found that medical students who participate in at least one year of rural clinical placements during their training have a greater likelihood of practising rurally than their city‐trained peers.^4^, ^5^, ^7^, ^8^, ^9^, ^10^, ^11^

Rural clinical schools (RCSs) provide the opportunity for medical students to undergo a clinical placement in a rural area in Australia. Other studies have found that students’ satisfaction with their RCS placement is also associated with their rural career intent.^12^, ^13^ This correlation might be due to high‐quality supervision and working with rural mentors.^12^ More recently, the idea of self‐efficacy has been proposed as a factor that supports rural career intent.^14^ Self‐efficacy can be described as the optimistic self‐belief in one's own competence or chances of successful accomplishment of a specific outcome.^15^ Rural practice self‐efficacy has been proposed as the mechanism by which the factors described above influence medical students’ intention to practise rurally.^14^ Among other factors, having generalist intentions prior to the completion of medical school has also been shown to be associated with rural career intent after graduation.^16^, ^17^

Most previous literature on medical students’ rural career interests have included all locations outside of a major or capital city.^3^, ^11^ Some more recent studies have started to reduce this gap in the literature by looking at factors influencing career interest in smaller regional and rural towns (Modified Monash Model 3‐7).^4^, ^8^, ^9^ Research to date has failed to explore medical workforce intent for remote and very remote areas in Australia. This study seeks to consider the cohort of students who spend one year in an RCS and compare those who are interested in remote and very remote practice with those students interested in practising in major cities and regional areas.

## 2 METHODS

This was a cross‐sectional study. The Federation of Rural Australian Medical Educators (FRAME) have developed an annual national exit survey for students who have completed a one‐year‐long RCS placement from 17 medical schools across Australia.^18^ This survey is used to obtain information about the students’ experience of their placement at the RCS. The survey includes questions about their clinical exposure, supports, personal well‐being and future career intentions. De‐identified data from this survey were used to gain a deeper insight into the RCS factors correlated with medical students’ interest in working in remote and very remote communities. We have analysed the FRAME data and included responses over a 5‐year period (2013‐2017). A chi‐square test was performed to ensure that there was no statistically significant difference in remote and very remote career intent between each year's survey, and the annual surveys were then aggregated to perform the analysis. There was a survey response rate of 80.3% over this 5‐year period.

### 2.1 Measurements

#### 2.1.1 Career intent

Students were asked to report on where they wish to practise upon finishing their training. They were asked to rank the following locations based on the ASGS‐RA classification from 1 (most preferred) to 5 (least preferred). RA1 was described as a major city of Australia; RA2 as inner regional Australia; RA3 as outer regional Australia; RA4 as remote Australia; and RA5 as very remote Australia.^19^ Participants who listed a major city of Australia (RA1) as 1 (most preferred) on the 5‐point Likert scale were categorised as having capital or major city career intent. Participants who listed inner regional Australia or outer regional Australia (RA2 or RA3) as 1 (most preferred) were categorised as having regional intent. Participants who listed remote or very remote Australia (RA4 or RA5) as 1 (most preferred) were categorised as having remote or very remote career intent. It is acknowledged that medical student remote and very remote career intent might not correlate with actual practice, but this was considered the best proxy using these survey data.

#### 2.1.2 Rural background

Students were asked whether they considered themselves to come from a rural background. This allowed students to consider their own rural identity, which we believe is an appropriate measure of rural background when considering their future career intentions. We acknowledge that this definition does not take into account the time spent living in a rural area. Any participants who answered ‘yes’ to this question were considered to have a rural background. Participants who answered ‘no’ were categorised as not having a rural background.

#### 2.1.3 Generalist intentions

Upon finishing their placement at an RCS, students were asked to rank their career preferences from 1 (most preferred) to 3 (least preferred) from the following 3 options: (a) general practice (FRACGP) or rural medicine (FACRRM); (b) generalist specialist; and (c) subspecialist/other. Participants who listed general practice or rural medicine as 1 (most preferred) on the 3‐point Likert scale were categorised as having generalist intentions. Participants who listed general practice or rural medicine as 2‐3 on the 3‐point Likert scale were categorised as not having generalist intentions.

#### 2.1.4 RCS experience increased interest in remote and very remote medicine

The following statement: ‘My RCS experience has increased my interest in pursuing a medical career in remote and very remote Australia (RA4‐5)’ was answered on a 5‐point Likert scale: strongly disagree, somewhat disagree, neutral, somewhat agree and strongly agree. If a student answered somewhat agree or strongly agree, it was judged that their RCS experience increased their interest in remote and very remote medicine. If a student answered neutral, somewhat disagree or strongly disagree, it was said that their RCS experience did not increase their interest in remote and very remote medicine. This variable captures changes in interest level between RCS entry and exit for RA4 and RA5 locations, independent of whether this change in interest alters first preference career intent.

#### 2.1.5 RCS placement satisfaction

Students’ satisfaction of their RCS placement was measured by the following statement: ‘I would recommend the RCS experience to other medical students’. This was answered on a 5‐point Likert scale: strongly disagree, somewhat disagree, neutral, somewhat agree and strongly agree. Students who answered somewhat agree or strongly agree were judged as satisfied with their RCS placement. Students who answered neutral, somewhat disagree or strongly disagree were judged as not being satisfied with their RCS placement.

#### 2.1.6 Rural‐based mentor

Students were given the statement ‘I have a rural based clinician as a mentor’ and asked to answer on a 5‐point Likert scale: strongly disagree, somewhat disagree, neutral, somewhat agree and strongly agree. Students who answered somewhat agree or strongly agree were said to have a rural‐based mentor. Students who answered neutral, somewhat disagree or strongly disagree were said to not have a rural‐based mentor.

#### 2.1.7 Rural practice self‐efficacy

Six questions were used to measure rural self‐efficacy:

- ‘Rural practice is too hard’
- ‘I have necessary skills to practice in a rural setting’
- ‘I get a sinking (anxious) feeling when I think of working in a rural setting’
- ‘I have a strong positive feeling when I think of working in a rural setting’
- ‘People tell me I should work in a rural setting’
- ‘I see people like me taking up rural clinical practice’

Each of the questions was answered on a 5‐point Likert scale, which was scored from 1 to 5. The negatively framed questions were scored in reverse order resulting in a score between 1 (strongly agree) and 5 (strongly disagree). The aggregate from these 6 questions was used as their rural self‐efficacy score, ranging between 6 and 30. The model used to calculate the students’ rural self‐efficacy score was designed by Isaac et al.^14^

Finally, 2 other variables were also analysed: sex and being an international fee‐paying student.

### 2.2 Data analysis

Data were analysed using the statistical package SPSS V. 23 (SPSS IBM). Descriptive data were examined to determine study variables. Cross‐tabulation, chi‐square tests and independent‐sample *t* tests were used as applicable to determine the factors associated with intent in remote and very remote locations. Multivariable forward stepwise logistic regression analyses were used to investigate the independent factors associated with remote and very remote career intent compared with capital or major city career intent. We repeated the multivariable analysis to investigate the independent factors associated with remote and very remote career intent compared with regional career intent. The models included sex, rural background, prior generalist intentions, the RCS experience increasing interest in RA4 and RA5, RCS placement satisfaction, rural‐based mentor and rural practice self‐efficacy. Statistical significance was defined at a value of *P* < .05.

### 2.3 Ethics approval

This study has ethics approval by the Flinders University Social and Behavioural Research Ethics Committee (project number 4098).

## 3 RESULTS

Responses from 3328 medical students were analysed over a period of 5 years from 2013 to 2017. Of the survey participants, 56.7% were female, 42.6% considered themselves to come from a rural background, and 7.0% of the students were interested in practising in a remote or very remote town in Australia (Table 1).

**Table 1:** Characteristics of the sample (n = 3328)

Variable	N (%)
Participants by year	
2013	668 (20.1)
2014	644 (19.4)
2015	643 (19.3)
2016	677 (20.3)
2017	696 (20.9)
Female sex	1886 (56.7)
Rural background	1417 (42.6)
International fee‐paying	99 (3.0)
Generalist intentions	916 (27.5)
RCS increased interest in RA4 and RA5	1342 (40.3)
RCS placement satisfaction	3029 (91.0)
Rural‐based mentor	1972 (59.3)
Interested in remote and very remote practice	234 (7.0)
Career intent	
Major cities of Australia	1250 (37.6)
Inner regional Australia	1188 (35.7)
Outer regional Australia	610 (18.3)
Remote Australia	191 (5.7)
Very remote Australia	43 (1.3)

Abbreviation: RCS, rural clinical school.

Table 2 illustrates the effect of various factors on career intent. There were a higher proportion of women and those with rural background indicating an interest in regional and remote or very remote practice. Being female sex (*P* < .001) and those considering themselves coming from a rural background (*P* < .001) were more likely to report an intent to practise in remote and very remote areas after graduation. Of those students with prior generalist intentions, 16.0% expressed an interest in remote/very remote practice compared with only 3.7% without prior generalist intentions (*P* < .001). Similarly, 12.8% of students who felt their RCS experience increased their interest in remote and very remote practice reported an intent to practice in remote and very remote areas upon graduation, and this compared favourably with the other students (3.3%, *P* < .001). Higher frequencies of remote and very remote medical practice intention were also associated with students who reported satisfaction with their RCS placement (7.4% vs 3.8%, *P* < .001), having a rural‐based mentor (5.4% vs 8.4%, *P* < .001) and having a higher rural practice self‐efficacy score (3.1% vs 7.2%, *P* < .001).

**Table 2:** Bivariate analysis of the effect of different variables on career intent (n = 3256)

Variable	Capital or major city N (%)	Regional N (%)	Remote/very remote N (%)	*P*‐value
Sex				
Male	603 (43.4)	712 (51.3)	74 (5.3)	<.001
Female	634 (34.1)	1071 (57.5)	157 (8.4)	
Rural background				
No	936 (51.1)	822 (44.8)	75 (4.1)	<.001
Yes	294 (21.0)	949 (67.8)	156 (11.2)	
International fee‐paying				
No	1204 (37.9)	1744 (54.9)	230 (7.2)	.299
Yes	39 (41.1)	53 (55.8)	3 (3.2)	
Generalist intentions				
No	1064 (45.4)	1194 (50.9)	86 (3.7)	<.001
Yes	174 (19.1)	590 (64.8)	146 (16.0)	
RCS increased interest in RA4 and RA5				
No	861 (44.5)	1010 (52.2)	63 (3.3)	<.001
Yes	377 (28.4)	781 (58.8)	170 (12.8)	
RCS placement satisfaction				
No	146 (62.1)	80 (34.0)	9 (3.8)	<.001
Yes	1087 (36.2)	1693 (56.4)	223 (7.4)	
Rural‐based mentor				
No	590 (45.4)	639 (49.2)	70 (5.4)	<.001
Yes	649 (33.2)	1144 (58.5)	164 (8.4)	
Rural practice self‐efficacy				
Score	21.28 (3.5)	24.0 (3.2)	25.4 (3.1)	<.001
Total	1239 (38.1)	1783 (54.8)	234 (7.2)	

Abbreviation: RCS, rural clinical school.

Table 3 first shows the factors associated with remote and very remote career intent when compared to capital or major city intent in a multivariable logistic regression analysis. Female sex (OR 2.0 [95% CI 1.3‐3.1]), rural background (OR 5.8 [95% CI 3.8‐8.7]), generalist intentions (OR 8.6 [95% CI 5.7‐13.0]), RCS experience increasing interest in remote and very remote practice (OR 4.6 [95% CI 3.0‐7.0]) and having high rural practice self‐efficacy (OR 1.4 [95% CI 1.3‐1.5]) were associated with career intent in remote and very remote locations when compared to capital or major city intent. RCS placement satisfaction (OR 0.6 [95% CI 0.2‐1.6]) and having a rural‐based mentor (OR 1.2 [95% CI 0.8‐1.8]) were not related to remote and very remote career intent after mutual adjustment of other factors in the model.

**Table 3:** Multivariable logistic regression analyses of the effect of different variables on remote and very remote career intent compared with capital or major city intent (n = 1377), and remote and very remote career intent compared with regional intent (n = 1902)

Variable	Remote and very remote intent vs major city intent	Remote and very remote intent vs regional intent
	Odds ratio (95% CI), *P*‐value	Odds ratio (95% CI), *P*‐value
Female sex	2.0 (1.3‐3.1), <.001	1.2 (0.9‐1.7), .17
Rural background	5.8 (3.8‐8.7), <.001	1.7 (1.3‐2.4), .001
Generalist intentions	8.6 (5.7‐13.0), <.001	3.0 (2.2‐4.0), <.001
RCS increased interest in RA4 and RA5	4.6 (3.0‐7.0), <.001	3.0 (2.2‐4.2), <.001
RCS placement satisfaction	0.6 (0.2‐1.6), .30	0.8 (0.3‐1.8), .56
Rural‐based mentor	1.2 (0.8‐1.8), .52	1.1 (0.8‐1.5), .53
Rural practice self‐efficacy	1.4 (1.3‐1.5), <.001	1.1 (1.0‐1.1), .005

Abbreviation: RCS, rural clinical school.

Table 3 secondly shows the factors associated with remote and very remote career intent when compared to regional intent in a multivariable logistic regression analysis. Rural background (OR 1.7 [95% CI 1.3‐2.4]), generalist intentions (OR 3.0 [95% CI 2.2‐4.0]), RCS experience increasing interest in remote and very remote practice (OR 3.0 [95% CI 2.2‐4.2]) and having high rural practice self‐efficacy (OR 1.1 [95% CI 1.0‐1.1]) were associated with remote and very remote career intent when compared to regional career intent. Female sex (OR 1.2 [95% CI 0.9‐1.7]), RCS placement satisfaction (OR 0.8 [95% CI 0.3‐1.8]) and having a rural‐based mentor (OR 1.1 [95% CI 0.8‐1.5]) were not associated with remote and very remote career intent.

## 4 DISCUSSION

This national study looked specifically at career interest in remote and very remote towns in Australia (RA4‐5) compared firstly with capital or major cities, and secondly with regional areas. The study shows that rural background, generalist intentions and having a high rural practice self‐efficacy are associated with medical students’ interest in practising in remote and very remote towns in Australia. These findings reinforce the importance of the medical school selection process in improving the medical workforce in remote and very remote areas.

It is known that RCS exposure is a critical factor in increasing interest in rural practice.^11^, ^20^ This study also validated the importance of the students’ experiences during rural placement on career intent in remote and very remote locations. Students who felt that their RCS experience increased their interest in remote and very remote practice were 3 times as likely to want to practise in these areas after graduation when compared to students with regional career intent. This number raised to more than 4 times as likely when compared to students interested in practising in capital or major cities. This implies that there are modifiable factors contributing to the RCS experience that can be targeted by RCSs to improve students’ remote and very remote experiences and increase medical students’ interest in remote and very remote practice. The FRAME study variables did not enable us to explore the curriculum, experiential and placement qualities, which contributed to this result. Further research is needed to tease these out.

Interestingly, most of the factors that were found to be associated with remote or very remote intent when compared to capital or major city intent were also consistently significant when students with remote or very remote intent were compared to those with regional intent. Despite the similarity in factors, it is noted that the influences of rural background and generalist intentions were more prominent when remote and very remote career intent was compared with major city career intent as opposed to when remote and very remote career intent was compared with regional intent. This large difference is shown by the non‐overlapping 95% CIs. Female sex was the only factor that became non‐significant for remote or very remote intent when compared to regional intent. This suggests that these factors that have been previously recognised as contributors to rural career intent are further associated with remote and very remote career intent.

Previous studies have found RCS placement satisfaction to be associated with medical students’ rural practice intentions.^12^, ^13^ Once accounting for potential confounding factors in both of our multivariable analyses, RCS placement satisfaction was not found to be associated with remote and very remote career intentions. This suggests that there are other more crucial aspects of the RCS experience that contribute to this interest in remote and very remote areas.

One of the strengths of our study is that we have obtained our data from the FRAME survey, which has 17 RCSs from across Australia participating. We have also used data over a 5‐year period, which enables us to consider associations that are likely consistent over time rather than an individual cohort effect. This has also allowed us to investigate the smaller population of medical students (7.0%) interested in remote and very remote practice. This adds value from other studies as instead of combining rural career intent as one large group, we were able to separate regional career intentions from remote and very remote career intentions.

One limitation of our study is that due to the nature of the FRAME survey, this study only investigates medical students who have spent one year at an RCS, which is a factor increasing rural career intent in itself.^11^, ^20^ A second limitation is that our outcome was remote and very remote career intent, which does not necessarily correlate with medical students actually practising in these areas throughout their career. This has been a recurring limitation in many similar studies and requires longitudinal follow‐up of medical students to determine their actual practice location.^21^ Our study did not take into account factors such as the location of the students’ RCS placement, and it would be interesting to see whether undergoing an RCS placement in a remote or very remote town is associated with interest in remote and very remote practice. As this was an annual survey, it is possible that students might have completed the survey multiple times if they participated in an RCS more than once. Due to the high survey response rate, it is unlikely that the results have been significantly affected by response bias.

The factors that we found to be associated with medical students’ interest in remote and very remote practice were similar to the factors that have been previously found to be associated with medical students’ interest in rural practice. The implications of this is that previous research in this field defining rural areas as all areas outside of a capital or major city might be appropriately extrapolated to these remote and very remote areas. RCS placement satisfaction was the only factor that has been previously found to be associated with rural career intent that was not shown to be associated with remote and very remote career intent in this study. Extending RCS placements to involve a larger number of remote and very remote towns might result in more medical students being interested in practising in these areas. Our study points to the need for future research that considers the RCS location rurality alongside medical student career intentions, which might provide a greater insight into this.

In conclusion, we found that rural background, prior generalist intentions, rural practice self‐efficacy and students who felt that their RCS experience increased their interest in remote and very remote practice were all associated with students wanting to practice in remote and very remote towns in Australia. Factors contributing to the RCS experience play a role in increasing interest in remote and very remote practice. RCS placement satisfaction was not found to be associated with remote and very remote career intentions. As Australia seeks to improve access to medical care for the population who live in remote and very remote areas, this study supports the need for further research into the relationship between RCS location and future career location, which might highlight a need for high‐quality longitudinal clinical placements for medical students in remote and very remote locations.

## CONFLICT OF INTEREST

DR is a medical student who attended the Flinders University Parallel Rural Community Curriculum. VI and LW each have leadership roles in rural clinical schools, which are funded through the Australian Government Rural Health Multidisciplinary Training grants.

## AUTHOR CONTRIBUTIONS

DR: Conceptualisation (support); data curation (lead); formal analysis (support); methodology (support); writing—original draft preparation (lead). VI: Formal analysis (lead); supervision (support); writing—review and editing (equal). LW: Conceptualisation (lead); methodology (lead); supervision (lead); writing—review and editing (equal).
